# Quadricuspid Aortic Valve With Severe Aortic Regurgitation

**DOI:** 10.1002/ccr3.70701

**Published:** 2025-07-31

**Authors:** Takumi Minatoya, Atsushi Hayashi, Hiroki Okamoto, Kohei Asada, Noriyuki Takashima, Tomoaki Suzuki, Yoshihisa Nakagawa

**Affiliations:** ^1^ Division of Cardiovascular Medicine, Department of Internal Medicine Shiga University of Medical Science Otsu Shiga Japan; ^2^ Division of Cardiovascular Surgery, Department of Surgery Shiga University of Medical Science Otsu Shiga Japan

**Keywords:** aortic regurgitation, aortic valve surgery, quadricuspid aortic valve, transesophageal echocardiography

## Abstract

Whenever a quadricuspid aortic valve (QAV) was present, it was historically only identified during open heart surgery. However, recent advances in transthoracic echocardiography have made it possible to detect QAV preoperatively. Further evaluation using transesophageal echocardiography or computed tomography before open surgery may help guide the surgical treatment strategy.

## 1 Case Report

A 76‐year‐old man presented to the emergency department with acute dyspnea and was admitted to our hospital with a diagnosis of acute heart failure. The patient had a history of hypertension, dyslipidemia, and severe aortic regurgitation (AR) with reduced left ventricular (LV) contractility and LV enlargement, but had discontinued his outpatient visits 3 years before this admission. On physical examination, his blood pressure was 110/79 mmHg, and cardiac auscultation revealed a III/VI diastolic murmur along the left sternal border. A chest radiograph showed a cardiothoracic ratio of 73% with a butterfly shadow in both lungs. Blood tests revealed an elevated brain natriuretic peptide level (2278 pg/mL). Transthoracic echocardiography showed severe AR, LV contractile dysfunction with an LV ejection fraction of 25%, and LV dilatation with an LV end‐diastolic diameter of 63 mm. Within a few days of admission, his status improved. Due to symptomatic severe AR with the severely reduced LV function and LV enlargement, aortic valve replacement surgery was recommended.

**FIGURE 1:**
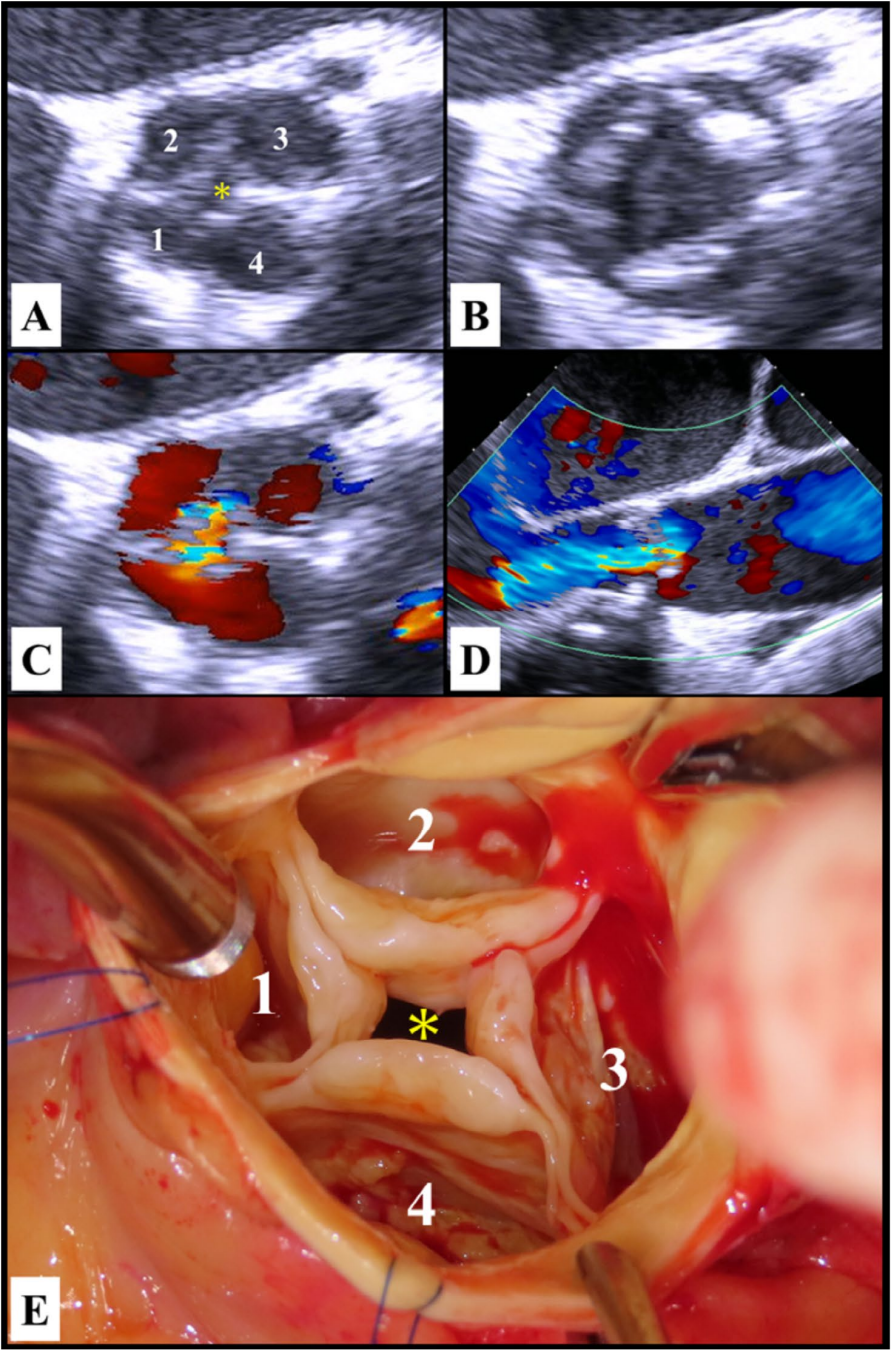
Preoperative transesophageal echocardiography showed four leaflets in the short‐axis view of the aortic valve (A, diastolic phase; B, systolic phase). Severe central aortic regurgitation (C, short‐axis view; D, long‐axis view) resulted from a small diastolic coaptation defect (A and E, asterisk). Intraoperatively, the surgeon's view revealed a quadricuspid aortic valve (E). The four cusps were of equal size and the accessory cusp was located between the right and left cusps (A and E, 1: Right‐coronary cusp, 2: Non‐coronary cusp, 3: Left coronary cusp, 4: Accessory cusp).

Preoperative transesophageal echocardiography in the short‐axis view showed that the aortic valve had four leaflets (Figure 1A,B), consistent with a QAV, and a central jet of AR (Figure 1C,D). Surgical findings revealed the presence of a QAV characterized by four equal‐sized cusps (Figure 1E). This configuration was classified as type A according to the Hurwitz and Roberts classification [1, 2]. A bioprosthetic valve was implanted. He had an uneventful postoperative course.

## Author Contributions

**Takumi Minatoya:** writing – original draft. **Atsushi Hayashi:** writing – review and editing. **Hiroki Okamoto:** writing – review and editing. **Kohei Asada:** resources. **Noriyuki Takashima:** resources. **Tomoaki Suzuki:** resources. **Yoshihisa Nakagawa:** supervision.

## Consent

Written informed consent was obtained from the patients to publish this report in accordance with the journal's patient consent policy.

## Conflicts of Interest

The authors declare no conflicts of interest.

## Data Availability

The data that support the findings of this study are available from the corresponding author upon reasonable request.
